# MiRNA Influences in Neuroblast Modulation: An Introspective Analysis

**DOI:** 10.3390/genes9010026

**Published:** 2018-01-09

**Authors:** Vanessa Zammit, Byron Baron, Duncan Ayers

**Affiliations:** 1 National Blood Transfusion Service, St. Luke’s Hospital, PTA1010 G’Mangia, Malta; vanessa.zammit@gov.mt; 2 School of Biomedical Science and Physiology, University of Wolverhampton, Wolverhampton WV1 1LY, UK; 3 Centre for Molecular Medicine and Biobanking, Faculty of Medicine and Surgery, University of Malta, MSD2080 Msida, Malta; byron.baron@um.edu.mt; 4 School of Health Sciences, Faculty of Biology, Medicine and Health, The University of Manchester, Manchester M13 9PL, UK

**Keywords:** miRNA, neuroblast, nsc, neuroblastoma

## Abstract

Neuroblastoma (NB) is the most common occurring solid paediatric cancer in children under the age of five years. Whether of familial or sporadic origin, chromosome abnormalities contribute to the development of NB and cause dysregulation of microRNAs (miRNAs). MiRNAs are small non-coding, single stranded RNAs that target messenger RNAs at the post-transcriptional levels by repressing translation within all facets of human physiology. Such gene ‘silencing’ activities by miRNAs allows the development of regulatory feedback loops affecting multiple functions within the cell, including the possible differentiation of neural stem cell (NSC) lineage selection. Neurogenesis includes stages of self-renewal and fate specification of NSCs, migration and maturation of young neurones, and functional integration of new neurones into the neural circuitry, all of which are regulated by miRNAs. The role of miRNAs and their interaction in cellular processes are recognised aspects of cancer genetics, and miRNAs are currently employed as biomarkers for prognosis and tumour characterisation in multiple cancer models. Consequently, thorough understanding of the mechanisms of how these miRNAs interplay at the transcriptomic level will definitely lead to the development of novel, bespoke and efficient therapeutic measures, with this review focusing on the influences of miRNAs on neuroblast modulations leading to neuroblastoma.

## 1. Introduction

Neuroblastoma (NB) is a paediatric cancer derived from neural-crest cells [1]. NB tumourigenesis commences from precursor cells of the sympathetic nervous system and typically occurs in the adrenal gland, sympathetic ganglia and paraganglia, or along the spinal cord [2]. However, the majority of NB tumours can be found in the abdomen, adrenal medulla, and other organs of the body involving the sympathetic nervous system [3].

### NB Manifestation and Prognosis

The majority of NB incidences are of sporadic origin and can be caused by multiple aetiologies, including chromosomal loss [4]. Prognostics of NB tumours are associated with major deletions on chromosome 11q [5,6], which in turn are associated with loss of 3p [7,8]. Other additional large-scale chromosome abnormalities include the loss of 4p, 9p, and 14q, and gain of 1q, 7q, 2p, and 11p, and all can contribute to the development of NB [9]. Furthermore, this inequity also contributes to miRNAs dysregulations [10]. 

Alternatively, albeit in the vast minority of NB cases, NB can occur due to familial origin and can be consequently traced at the genetic level along family trees [11]. Familial NB is typically linked with specific gene mutations such as anaplastic lymphoma kinase (ALK) [11] and, in the minority of such cases, mutations in the Paired-like homeobox 2b (PHOX2B) gene [12].

Classification of NB tumours is performed through globally recognised NB classification protocols such as the constantly updated guidelines presented by the International Neuroblastoma Risk Group (INRG) [13]. Classification of each individual NB clinical case is based according to the localisation and extent of the primary tumour, the absence or presence of metastasis, the extent of the disease at diagnosis, and other risk factors defined through imaging [14,15]. Biological and genetic heterogeneity play an important role in the clinical manifestation of NB. Consequently, genetic and molecular findings play major roles in NB treatment selection [16,17,18,19,20,21].

## 2. Biological Function of miRNAs

MicroRNAs (miRNAs), are a family of small, non-coding single stranded RNAs (ncRNAs) approximately 22nt in length, and miRNA-coding regions are located in introns or intergenic chromosomal loci [22].

In humans, miRNAs perform their gene regulatory functions, not though target transcript degradation, but through inducing a steric hindrance obstruction (following miRNA attachment on target transcript) for ribosomal machinery function in the process of mRNA translation [23]. Another mechanism for how miRNAs interact with the transcription/translation mechanism is through miRNA processing mechanisms of longer transcripts that are consequently integrated into the RISC complex. The absorbed miRNAs will regulate gene expression by directing the RISC complex towards the target transcript. However, further elucidation is required in order to understand the actual mechanism of how the RISC complex is guided [24].

MiRNAs regulate gene expression at the post-transcriptional level by targeting messenger RNAs (mRNAs), typically at the 3′ untranslated regions (UTRs) of their mRNA targets [25,26,27,28,29]. This regulation is executed by repressing translation [23] or decreasing transcript stability [30,31] and alternatively causing mRNA degradation, with a consequent inhibiting effect on downstream protein expression, therefore affecting cell proliferation, differentiation, apoptosis or other biological processes [27].

Most human genome component genes are regulated by miRNAs [32,33], and it is advocated that small ncRNAs have the potential to regulate all human genes [34]. Dysregulation of ncRNAs is a fulcrum of several diseases [35,36].

MiRNAs are crucial elements involved in the mechanisms of cell cycle, proliferation, differentiation, apoptosis, and metabolism. Dysregulated miRNAs can lead to cancer development, cancer stem cell formation, autophagy, multidrug resistance, epithelial-mesenchymal transition (EMT), migration, invasion, and metastasis [37,38,39]. MiRNAs can also have important tumour suppressor properties. Epigenetically inactivated tumour suppressor miRNAs can contribute to NB pathogenesis [40]. These miRNAs are downregulated by MYCN amplification. The LIN28B situated on the LIN28B-let-7-MYCN axis, can downregulate let-7, a tumour suppressor miRNA family that causes an up-regulation of MYCN, resulting in NB growth stimulation [41]. MYCN can down-regulate the epigenetically controlled miR-335, a tumour suppressor miRNA, leading to over-expression in target genes of the Transforming Growth Factor beta (TGF-β) non-canonical pathway, inhibiting both the migration and invasion of NB cells [42].

## 3. The Role of miRNAs in Neural Lineage Differentiation

### 3.1. Development of the Neural Lineage

The neural lineage may evolve from stem cells and progenitor cells derived from the neuroepithelium, and both eventually differentiate into one of the three cell types that make up the nervous system, namely neurones, astrocytes, and oligodendrocytes. Neural progenitors undergo a further differential step and develop into neurones and glia [43]. MiRNAs interact with different signalling pathways, influencing expression of various transcripts that, in turn, interact with their targets to create regulatory feedback loops affecting multiple functions within the cell, thus modulating the differentiation and lineage of neural stem cells (NSCs) and their progeny [44].

Neurogenesis is a well-maintained process [45] that includes stages of self-renewal and fate specification of NSCs, migration and maturation of young neurones, and functional integration of new neurones into the neural circuitry. MiRNAs regulate these stages by base-pairing with target mRNAs and therefore control target gene expression [46]. Furthermore, miRNAs modulate post-mitotic neural cell production during the development of the central nervous system (CNS), where a balance between proliferation and differentiation of neural progenitors is achieved [47]. Although miRNAs are involved in a variety of neurogenesis processes and neural identities, specific subsets of these miRNAs have different mechanisms for regulating neural stem cell proliferation and differentiation (neurogenesis-related miRNAs) and neuronal identity (neuronal-specification-related miRNAs) [48].

### 3.2. miRNA Regulation

Furthermore, various studies have elucidated the role played by mature miRNAs in manipulating the expression of stem cell regulators responsible for stem cell fate determination and self-renewal [49,50,51,52]. The CNS expresses large amounts of miRNAs, which makes current knowledge on miRNA regulation during neurogenesis very limited. However, a deeper insight is now possible due to an increase in neural cell types derived from human pluripotent stems cells and the possibility of generating specific neural subtypes [53]. NSCs are described as undifferentiated precursors with self-renewing properties that retain the ability to differentiate into glial (astrocytes and oligodendrocytes) and neuronal lineages [49,54,55,56,57] and can be found in both adult and the developing CNS [58,59]. Brain development and neural differentiation of stem cells are both regulated by the miRNAs expressed in the CNS, which indicates the important role miRNAs play in neural development and function [60,61,62,63,64,65]. MiRNAs are involved in neural induction, progenitor expansion, differentiation and neuronal subtype specification [66,67], regulate neuronal migration [68,69], and are associated to neuronal function, neurite outgrowth and synaptic plasticity [70,71]. Described below is a selection of miRNAs that are recognised to influence in such manners.

### 3.3. Hsa-miR-765

miR-765 regulates expression of the hairy and enhancer of split-1 (Hes1) gene, and stimulates NSC proliferation, and when this is overexpressed the expression of ki-67 and β-tubulin-III are increased while Glial fibrillary acidic protein (GFAP) expression is inhibited [72]. The Hes gene encodes basic helix-loop-helix (bHLH) transcriptional modulators [73,74] and is highly expressed in the CNS. In addition, it acts as a downstream modulator of the Notch pathway [75,76]. MiR-765 plays a major role in the development of the CNS and also regulates both the differentiation and proliferation of NSCs [77,78,79]. NSCs will differentiate into neurones when modulation of Hes1 occurs via miR-9 [57]. Inhibiting Hes1 with miR-381 will also affect the differentiation and proliferation mechanism of NSCs [80]. MiR-765 is involved in various diseases [81,82,83,84], can act as a tumour suppressor miRNA [82], and can also regulate arterial stiffness [83]. MiR-765 furthermore targets the 3′-UTR of Hes1 and decreases its expression in NSC, and an overexpression of Hes1 decreases miR-765-induced proliferation of NSCs and inhibits neuronal differentiation [72].

### 3.4. Hsa-miR-146

MiR-146 miRNA regulates inflammation and other mechanisms related to the innate immune system [85]. Up-regulation of miR-146 is executed via inflammatory factors such as interleukin 1 and tumour necrosis factor-α. This consequently dysregulates target receptors of the innate immune system cytokine response [86], thus activating a feedback system which regulates the inflammatory response [87]. The Notch signalling pathway is extensively used in mammals during embryonic development and homoeostasis in adults [88]. Furthermore, Notch signalling is also necessary for the upkeep of neural stem cells and their progenitors, together with glial cell formation [89,90]. A family member of miR-146, miR-146a directly regulates the expression of Notch 1, limiting the formation of glioma stem cells and growth [91].

## 4. Role of miRNA in Neuroblastoma

NB development depends on both gene expression and the major role played by miRNAs as key effectors. Cancer epidemiology has shown that miRNAs can be found on both genomic regions and fragile sites involved in characterisation of the disease [92]. In addition to this, both loss-of-function and gain-of-function experiments in human cancer cells have confirmed that miRNAs are key players in all cancer stages, i.e. initiation, progression and metastasis [93,94].

Tumour cell behaviour varies according to the cancer model involved. In the presence of cancer, miRNAs function depends deeply on the mRNA targets and subsequently causes them to act as tumour suppressors or as oncogenes [95]. Conversely, other studies indicate that when compared to normal cells, miRNA expression in tumour cells is suppressed, which leads to the thought that miRNA biogenesis might be impaired in cancer [96,97]. Dysregulated miRNA expression can be induced by various mechanisms such as translocation, deletion, point mutations, and rearrangements. This therefore plays an important role in miRNA tumorigenic roles [98]. As in the case with other cancers, the aberrant expression of oncogenic miRNAs has a decisive role in the maintenance of neuroblastoma stem cells and tumorigenesis [99,100,101].

Regulated gene patterns are the fulcrum of neurogenesis [102,103] and it has been widely proven that miRNAs play an important role in the regulation of neural development and function [103]. Neuro-specific transcription activates cell cycle exit during differentiation, where miRNAs influence the functional and morphological specialisation of the cells by targeting both epigenetic factors and transcriptional repressors [43]. During neuronal differentiation, miR-124 also regulates alternative splicing by targeting polypyrimidine tract-binding protein 1 (PTBP1) mRNA, a repressor of neuron-specific pre-mRNA splicing, leading to the activation of neuronal gene expression [104]. MYC is another transcription factor that regulates miRNA [105,106] transcription, as does the tumour suppressor gene Tumour Protein P53 (TP53) [107].

The role of miRNAs and their interaction in cellular processes is a new and developing field of cancer genetics. Experimentation with expression variation and selective inhibition of miRNAs has helped elucidate the functional roles of these regulators which, in turn, has helped in establishing protocols for deterring cancer progression and improve patient prognosis. The clinical picture of NB is usually characterised based on the type of genetic abnormality [3] and progression of the epigenetic changes [108]. Conversely, deregulation of miRNA has helped classify both the pathogenesis and heterogeneity of NBs [109,110]. Subtypes of NB tumours exhibit heterogeneity, according to the genetic abnormality present, subsequently determining the clinical outcome in the individual NB patient [3]. What underlying mechanisms contribute to NB pathogenesis, metastasis and apoptosis is not yet fully understood, which leads to multiple difficulties in the identification of novel biomarkers or therapeutic targets for early detection or treatment [111].

### 4.1. MicroRNA Let-7 Family

The miRNA let-7 family (let-7), consisting of 12 members [112], is present in multiple genomic locations [113]. Let-7 targets cyclin D1, causing overexpression and leading to the promotion of cell cycle exit and differentiation [114]. Let-7 also suppresses the differentiation of human embryonic cell neural progenitor cells [115], regulates NSC self-renewal mechanisms [116] and determines cell fate [117,118]. Overexpression of let-7a directly affects both proliferation and differentiation of NSCs, though further elucidation is required to understand the mechanism of action [119,120]. Agmatine, an endogenous amine produced by decarboxylation of L-arginine by arginine decarboxylase [121], can enhance NSC differentiation into neurones by modulating expression of let-7a [122]. It also regulates neuroprotection and increases neuronal differentiation in NSCs by activating extracellular signal-regulated protein kinases 1 and 2 (ERK1/2)—members of the mitogen-activated protein kinase super family—and can mediate cell proliferation [123]. Agmatine promotes ERK activation by negatively regulating let-7a such that, if it is overexpressed, leads to decreased ERK phosphorylation [122].

Lin28B has been associated with various tumours, including paediatric cancers [124,125,126]. Small nucleotide polymorphisms (SNPs) in the Lin28B gene predispose the development of NB and elevated Lin28B expression levels are associated with a poor prognosis [127]. Lin28B mechanistic function during development of the sympathetic nervous system is still not clear [128]. However, overexpression of Lin28B promotes NB tumorigenic characteristics in postnatal sympathetic ganglia and adrenals [41]. Lin28 and Let-7 expressions are regulated by each other [125,129], which consequently indicate that these can have similar functions in relation to the development of the nervous system [130,131].

### 4.2. MicroRNA-124

MicroRNA-124 (miR-124) is a highly expressed tissue-specific [132,133,134,135,136] miRNA of the nervous system. In order to maintain the neural state, miR-124 downregulates the expression of non-neural mRNAs by repressing the expression of non-neural transcripts and thus directing the gene expression profile towards the neural state [60,104,137]. Upon cell lineage commitment, gene expression alteration is initiated by activating the expression of neuron-specific genes and repression of other genes that are not of the neuronal state. This alteration further enhances the important role that miRNAs play in regulating the gene expression in neuronal differentiation. High levels of miR-124 found in differentiated neural cells are associated with the absence of the transcription factor human specificity protein 1 (Sp1), indicating that Sp1 is a direct target of miR-124 [138]. Sp1 is essential in many cellular processes such as cell cycle progression, angiogenesis, and cell migration and invasion [139]. Sp1 is responsible for the regulation of multiple genes including vascular endothelial growth factor, Cyclin A2, Cyclin D1, E-cadherin, Phosphatase 2 A, and Matrix metalloproteinase [140]. A reduction in Sp1 expression inhibits mitosis [141] and is nearly absent in differentiated neurones [142], indicating that Sp1 expression is inhibited post- neuronal differentiation [143].

### 4.3. MYCN

MYCN is a helix-loop-helix leucine zipper transcription factor found in the peripheral and central nervous system [144]. This is encoded by the MYCN gene and affects the expression of long ncRNAs, which may contribute to oncogenic transformation and metastatic aggressiveness in NB [145]. MYCN amplification and cell heterogeneity are two factors that contribute to patient survival. MiRNA dysregulation in malignant NB is caused by MYCN amplification, chromosomal deletion, or abnormal epigenetic regulation [110,145]. In fact, various studies have focused on the role of miRNAs in association with MYCN amplification, chromosomal imbalances, prognosis and retinoic acid (RA)-induced differentiation [110,146,147,148] and have shown that chromosomal imbalances dysregulate miRNAs, which contribute to NB pathogenesis and tumourigenicity. MYCN binds to the promoter region of the genes responsible for the regulation of miRNA expression [149]. MiR-17-92 cluster members that are associated with NB tumourigenicity are also related to MYCN amplification. MiRNAs are also involved in RA-induced differentiation, and since RA is used in the treatment of NB, miRNA modulation can regulate the key genes involved in differentiation survival and the tumourigenic properties of NB [147].

MYCN amplification can be observed in approximately 25% of NB cases [150]. It is the most important oncogene [1] when dealing with NB, and the related sub-types presenting this oncogene have a high prediction of poor clinical outcome and metastatic disease [151]. MYCN interacts with various promoter regions of both genes and non-coding RNA sequences, and as a result of this, modifies their transcriptional activation [152,153,154,155], thus promoting tumour growth [156]. It functions by dysregulating the genes involved in the key pathways that affect NB [157]. The gene encoding the MYCN transcription factor is aberrantly repeated at chromosome 2p24, resulting in the amplification of the gene which, together with other chromosomal abnormalities, including 1p36 deletion, 11q deletion, and 17q gain [151,155,158], causes progression to advanced stages, aggressive metastasis, and consequently poor prognosis [159,160,161]. NB subtypes presenting MCYN amplification together with abnormalities at chromosome 1p and gain of 17q have the worst prognosis and are the major genetic subtype of metastatic NB [6,151,162].

**Figure 1:**
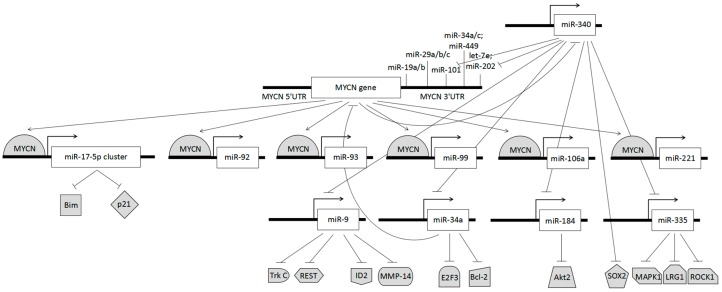
Dysregulation of microRNAs (miRNAs) and protein expression following MYCN amplification.

As shown in previous studies, expression levels of the MYCN gene are closely interlinked with an abnormality in miRNA regulation patterns [163,164,165,166] (Figure 1). In the presence of MYCN amplification several miRNAs, including miR-17-5p, miR-92, miR-93, miR-99, miR-106a, and miR-221, are upregulated. Malignant NB growth in the presence of MYCN amplification is correlated to the transactivation of the miR-17-5p-92 cluster [167,168,169]. This cluster inhibits translation of the cell cycle negative regulator cyclin-dependent kinase inhibitor 1 (p21) and Bcl-2-like protein 11 (Bim), a Bcl-2 interacting mediator of cell death [170]. By targeting this miRNA cluster using antago-miR-17-5p, p21, and Bim can be de-regulated, therefore inhibiting cell cycle processes and favouring apoptosis respectively, ultimately developing a treatment for counter growth of therapy-resistant malignant NB [170].

MYCN amplification is also responsible for the loss of chromosomal 1p36 heterozygosity, which downregulates the expression of miR-340, a miRNA that possesses tumour suppressant properties, and is upregulated by demethylation of an upstream genomic region [40]. This occurs during the process of neuroblastoma cell differentiation, and is induced by all-trans retinoic acid (ATRA) [40]. This deviant DNA methylation causes damping of miRNA tumour suppressors such as let-7, miR-101, and miR-202 that target MYCN; miR-9 that targets tyrosine kinase (Trk) C, RE-1 silencing transcription factor (REST), DNA-binding protein inhibitor (ID2), and Matrix metalloproteinase-14 (MMP-14); miR-34a that targets E2F transcription factor 3 (E2F3), B-cell lymphoma 2 (Bcl-2) and MYCN; miR-340 that targets SRY (sex determining region Y)-box 2 (SOX2); miR-184 that targets v-akt murine thymoma viral oncogene homolog 2 (Akt2); and miR-335 that targets Mitogen-Activated Protein Kinase 1 (MAPK1), leucine-rich repeat 1 (LRG1), and Ser/Thr Rho kinase 1 (ROCK1) [101] (Figure 1).

### 4.4. Tyrosine Kinase

Studies have shown that the Trk receptor family, consisting of TrkA, TrkB and TrkC, plays a relevant role in NB prognosis [171]. NB tumours expressing TrkA have a positive prognosis due to a higher chance of spontaneous regression or differentiation of the tumour as a result of the interaction of TrkA and nerve growth factor (NGF) [172]. Conversely, NB cells with low TrkA expression levels tend to become more invasive [156]. MiR-17-92a is associated with regulation of TrkA in view that both proliferation and migration of NB cells are affected in the presence of miR-17-92a [173]. However, the actual regulatory mechanism and signalling pathway for miR-17-92a and TrKA interaction require further elucidation in order to be able to implement NB treatment via miR-17-92a [173]. NB tumours expressing TrkB have a less favourable prognosis as the MYCN gene is amplified in this type of NB and also because TrkB ligands enhance viability, drug resistance and angiogenesis [172]. The mechanisms responsible for inducing drug resistance in NB are related to the presence of high levels of anti-apoptotic protein Bcl-2 and TrkB receptors that activate the PI3k/Akt pro-survival pathway. The tumour suppressor miR-204 is capable of targeting the 3′UTR of Bcl-2 and TrkB mRNAs, which means that transfecting malignant cells with these mimics will intensify sensitivity to cisplastin and etoposide, promoting apoptosis [174].

## 5. MiRNA Modulation of Stem Cells

Over the years, the important role played by miRNAs in stem cell fate determination and differentiation has become more pervasive. With ongoing research, novel miRNA/stem cell-related functions are being discovered, leading to the development of miRNA-based techniques applicable to therapies in regenerative medicine. It is well understood that cells can be reprogrammed using miRNAs, however further elucidation is required on whether miRNAs alone can actually induce reprogramming or whether they are improving the efficiency of reprogramming factors [175].

Another key aspect of miRNA modulation is in identifying the roles of circulating miRNAs and how these may be made to good use as part of miRNA therapeutic applications. This would be made possible by means of developing techniques which mimic stable exosomal miRNAs capable of efficiently delivering therapeutic miRNAs, effectively providing control on the proliferation and differentiation of stem cells used as the basis for tissue regeneration [176]. 

MiRNAs are involved in the simultaneous regulation of cell cycle exit and cell fate. This is exemplified by the cell cycle exit and differentiation promoted by overexpression of let-7 [114] or cell proliferation stimulated by the loss of miR-9 via an increase in expression of its target HES1 and consequently downregulation of its target p27, a cell cycle inhibitor [177]. Cell fate determination is clearly illustrated by the inhibition of transcription factor SOX9 by miR-124, encouraging the neurogenic precursor transition to neuroblasts [178] and the targeting by miR-219 and miR-338 of the transcription factors SRY-Box 6 (SOX6) and Hes family bHLH transcription factor 5 (Hes5), both involved with progenitor proliferation and stemness during oligodendrocyte differentiation [179,180].

### 5.1. Modulation of Stem Cells into Neuroblasts

Neuroblasts normally mature well before birth. However, occasionally not all cells mature completely and these gather to form small clusters in the adrenal glands. These neuroblasts eventually either die or mature into nerve cells, but in some instances, these continue to grow and develop into tumours that propagate to NB clinical conditions [181]. NB is generated from the sympathoadrenal lineage of the neural crest during development [182]. MiRNAs are major players in the development of the nervous system as these control differentiation, proliferation, self-renewal and regulate neurogenesis as summarized in Table 1.

Neuroblasts are essentially neural precursors that differentiate from neural stem cells and are committed to develop into neurons. These precursors may undergo three distinct dividing stages. The first stage is that of symmetric proliferation, which is encountered during early neural development. In this stage, the neuroblast divides yielding two identical cells. Midway through neural development, the cell divides to produce one precursor cell and another that is a transit-amplifying cell [196]. This phase allows the formation of neurons while still having precursors available to produce neurons and glia at a later stage. The self-renewal of neuronal precursors is promoted by Partitioning defective (Par) proteins [197], and downregulation of these proteins stops neurogenesis. Such regulation is under the control of a balance between mRNA translation and degradation by miRNAs [26]. The absence of miR-219 will keep precursors in their proliferation stage, which indicates that this miRNA promotes cell cycle exit and differentiation [198]. Finally, precursors will differentiate into either neurons or glia, ending neurogenesis and gliogenesis respectively [198]. These three stages are the primary key regulators of brain development.

The major signalling pathways of interest with respect to miRNA control, involved in the development of the nervous system are Wnt [199,200,201], Notch [202,203,204] and Sonic hedgehog (Shh) [205,206,207]. The Wnt signalling pathway facilitates the transition between neuroblast proliferation and neuronal differentiation via the beta-catenin pathways [208]. Notch signalling has an impact on several cellular processes including cell proliferation, differentiation and apoptosis [202,203,204]. Notch plays an important role in the maintenance and differentiation of NSCs. It promotes cell cycle exit, subsequently decreasing the number of neural progenitors [209]. Shh regulates the development of the nervous system in terms of ventral forebrain neuronal differentiation, midbrain dopaminergic differentiation, and cerebellar neuronal precursor proliferation [205,206,207]. In addition to these functions, it promotes self-renewal and proliferation of adult NSCs and regulates cellular migration.

Growth factors have also been implicated in the regulation of neurogenesis. Genes of interest are fibroblast growth factor-2 (FGF-2), insulin-like growth factor-1 (IGF-1), and vascular endothelial growth factor (VEGF), with all three binding to a ligand-specific receptor belonging to the Trk family [210]. FGF-2 encourages neural progenitor proliferation [211], IGF-1 signals neuronal differentiation of progenitor cells [212] and VEGF, a multifunctional growth factor, inducing mitosis in neural progenitor cells [213]. Bone Morphogenetic Proteins (BMPs), multifunctional extracellular signalling molecules forming part of the TGF-beta superfamily [214], are responsible for cell survival, proliferation and fate specification [215,216]. This signalling mechanism promotes glial differentiation and inhibits neuronal fate specification [217,218], prevents neuronal differentiation of Type B and C cells, supports neuroblast survival during migration [218] and increases NSCs proliferation, hence supporting neurogenesis [219]. 

Once stem cells are directed towards the neural lineage, a complex network regulates the self-renewal, proliferation, differentiation and spatial distribution of neuronal progenitors. In this regard, miR-134 and miR-184 have both been associated with neural progenitor maintenance and proliferation [66]. Neural induction is repressed through action on the BMP/TGFβ signalling pathway, blocking the transition of stem cells towards the neural lineage, via targeting by miR-302/367, increasing BMP signalling [220]. Similar effects result from the targeting of BMP repressors by miR-371 [221], transcription factor ZEB (a negative regulator of BMP/TGFβ signalling) by miR-200, or the transcription factor PAX6 by miR-96 [222]. Conversely, neuronal differentiation can be brought about by miR-125a/b and miR-135b blocking BMP signalling [223], miR-124, and miR-9 via the targeting of several components of the Notch signalling pathway, which in turn regulates neuronal development and expansion of neural progenitors [195,224,225], or the activation of PAX6 by miR-135b, promoting neural lineage entry [226].

### 5.2. Modulation from Neuroblasts into Neurones

During the stages of neural development, neural progenitors form different subtypes that differ in terms of neurotransmitter phenotypes, functions and innervation targets, and miRNAs contribute to the formation of such diversity [195]. According to their position in the neural tube, NSCs are subject to morphogens such as Shh, FGFs, Wnts and BMPs [227] which, when combined, initiate transcriptional programming of the neural subtypes [228,229]. MiRNAs also play a role as they modulate these morphogens by targeting their respective signalling cascades [44,230].

The subventricular zone (SVZ), the largest germinal region of the mammalian brain, acts as a neurogenic niche for stem cells from which neuroblasts will be produced [231]. MiRNAs are expressed at various stages of the SVZ stem cell lineage, including miRNA-124 that ends up highly expressed in the adult brain [232]. Over-expression of miRNA-124 in Hela cells promotes neurogenesis [137] Conversely, inhibiting miRNA-124 expression in cultured neurons induces non-neuronal transcript expression [233]. This miRNA targets PTBP, a repressor of neuron-specific splicing 11 and SCP1, a component of the REST transcription repressor complex [186]. However, contradicting studies [102,186] have shown that the actual role played by miRNA-124 during neurogenesis in vivo needs further elucidation. 

Amongst the primary functions of the Forkhead transcription factor family O (FoxO) are the maintenance of adult stem cells and long-term regeneration process implemented during organismal aging [234]. These two functions of FoxO are applied in the nervous system in relation to maintenance of adult neural stem cell reserves and neurogenesis [234,235]. MiRNAs that affect the mechanisms of FoxO will in turn determine the fate of neural stem/progenitor cells (NSPC) responsible for the evolvement of new neurons. There are several miRNAs involved in mechanisms that contribute to NSPC differentiation [103,236,237,238]. Amongst these miRNAs, Let-7b suppresses Hmga2, which promotes self-renewal, resulting in the inhibition of NSPC proliferation and an increase in neural differentiation [239]. Conversely, miRNA-9 encourages the proliferation and migration of NSPCs [183], and is also responsible for the regulation of the FoxO1 expression generated during neurogenesis of NSPCs that per se is downregulated during these early stages. Gain or loss of function of the FoxO1 expression ultimately leads to the inhibition or enhancement of NSPC differentiation [234]. MiRNA-25 promotes both NSPC proliferation and neuronal differentiation by regulating FoxO3 [190].

The miRNA-8/miRNA-200 family is another important regulator of neurogenesis [240] directed towards neural cells within neurogenic niches, and specifically direct the differentiated glial cells or neural stem/progenitor cells respectively [188,241,242,243]. The miRNA-8/miRNA-200 miRNA family members are considered to be tuning miRNAs, as they are co-expressed with their mRNA target [240], and their primary role is to control survival, proliferation, and cell cycle exit, and direct precursor cells to neuroblast cells and their subsequent differentiation into mature neurons [188,242,243]. Targets of these miRNAs include Sox2, E2F3 and TGFa which, when downregulated, initiate cell cycle exit and commitment of neural precursor cells to neuroblastic cells [188,243]. Premature differentiation is negatively regulated by miRNA-200, which suppresses LFNG, ZEB1 and SRF expression in the cells [241,242], and miRNA-8 regulation atrophin/RERE, and/or E2F3 protein levels ensure survival of neural stem/progenitor cells [188,244].

## 6. Application of miRNAs to Treatment and Management

Researchers in the field of diagnostic and prognostic biomarker discovery have been keenly searching for any biomolecule, be it RNA, DNA, protein, or modification thereof, which could hint towards any changes early in the development of neurological diseases or malignancies. Currently, brain tumours are diagnosed based on symptoms and neuro-imaging abnormalities, both of which appear during late stages in the pathogenesis [245]. This makes it extremely difficult to successfully treat aggressive malignancies which have spread throughout the brain (making the tumour inoperable) or have metastasised to other parts of the body. Changes to the cellular regulatory mechanisms involved in neural development and maintenance (as exemplified by Wnt, Notch and Shh) [246] begin much earlier and possessing the ability to detect some of these changes with confidence would lead to pre-symptomatic disease detection and diagnosis [247]. This in turn would allow for much more effective treatment, at a stage where tumours would have not yet accumulated so many mutations or produced a wide variety of sub-populations, improving the overall prognosis.

In NB, miRNAs have been widely studied as potential biomarkers for diagnosis and prognostic prediction, by looking both at the changes in the miRNA levels themselves and their downstream targets [109,248]. In order to apply these findings to patient screening and making the procedure less invasive, closer to routine testing and cost-effective, circulating miRNAs in body fluids such as plasma, serum and urine have been the preferred biomolecule cohort, which have also provided great advances in NB studies [249]. Serum miRNAs were shown to be much more stable than other biomolecules, being extremely tolerant to repeated freeze-thawing cycles and extremes of pH [250]. This is a great advantage for increasing the robustness and reliability of miRNA tests for patient screening, particularly if such testing cannot be performed immediately after sample collection.

The identification of potential prognostic biomarkers would help to distinguish or possibly even predict the point of transition from favourable to high-risk metastatic NB, which in turn further enhances the role of miRNAs in the monitoring of tumour progression [251], since levels of miRNA expression are altered by tumour cell engraftment and treatment—including drug-specific treatment. Consequently, decision-making and treatment selection would greatly benefit from such prognostic markers. The first miRNA to be identified as a putative tumour suppressor in NB was miR-34a which targets transcription factors and other genes responsible for cell proliferation [25]. Despite not being specific to NB, it provides an excellent indication of the pathways that are aberrantly activated.

Following from miRNA patient screenings and the mechanistic knowledge of their involvement in NB, a substantial amount of miRNA research has focused on their use as genetic modulators for the regulation of cancer development. One approach aimed at controlling the growth of malignant NB is that of stimulating tumour suppressor miRNAs through the design and administration of synthetic miRNA mimics or miRNA vectors with either a pre-miRNA sequence or an artificial miRNA hairpin sequence, which modulate the overall level of mature miRNA in malignant cells, producing a better prognostic outcome, either by promoting differentiation or apoptosis. A study has shown that in NB cell lines expressing the 1p36 hemizygous deletion, which have been transfected with miR-34a mimics showed degradation in the mRNA of Bcl-2 and MYCN, causing cell cycle arrest and apoptosis [252]. Another study indicated that when miR-184 was overexpressed using pre-miR-184, it inhibited the mRNA of Akt2, thus affecting the phosphatidylinositol 3-kinase (PI3K) pro-survival pathway and reducing tumour growth [253]. In addition, it has been demonstrated that miR-128, a tumour suppressor miRNA which is upregulated during RA-mediated differentiation of NB SH-SY5Y cells, will inhibit the expression levels of Reelin, a glycoprotein that acts as a guide during migration, and DCX—doublecortin located on chromosome X—a microtubule-associated protein essential for neuroblastic migration - limiting cell motility and invasiveness [254]. All these are potentially applicable therapies with required improvements in micro-delivery and targeted expression technologies.

## 7. Conclusions

Research conducted thus far has shown the importance of understanding the mechanisms controlling miRNA biogenesis as well as the conditions that disrupt these mechanisms, besides the search for and identification of potential miRNA biomarkers. This knowledge would also be key in restoring dysregulated miRNA expression profiles by means of small pharmacological agents, bypassing the challenges mentioned above of how to administer synthetic miRNA mimics or antagomiRs [255]. Furthermore, anti-tumour properties may be enhanced by suppressing the expression of oncogenic miRNAs and increasing the expression of tumour suppressor miRNA [256]. The NB-specific link of miRNA expression and the ability of miRNAs to possess both an oncogenic and suppressive nature enhances their importance among other putative biomarkers as potential therapeutic targets [257].

It is widely agreed that miRNAs are responsible for neural induction, neuronal differentiation and fate specification and so have thus have begun to develop into a translational medicine-based therapeutic approach for many conditions, including NB. The identification of additional putative miRNAs responsible for the neural development will further elucidate the mechanisms of action involved in both the physiological and pathological processes of the CNS.

## Figures and Tables

**Table 1:** miRNA involvement in neuronal development.

miRNA	Target	Expression	Role	Source
miR-9	Hes1	+	Down-regulates neural stem cell differentiation	[57]
	Stathmin REST			[183,184]
miR-29a	REST	+	Promotes neural differentiation	[185]
miR-124	PTBP1	+	Promotes neural differentiation	[186]
miR-124	SCP1	+	Promotes proliferation of neuronal precursors	[178]
	DLX-2			
	Jagged-1			
	SOX9			
miR-125	SCNBA EPHB2	+	Promotes neural differentiation	[187]
	KCNQ2			
	FLNA			
	SYN2			
	NEFM			
miR-200 family	SOX2	−	Promotes differentiation into neurons	[188]
	E2F3			
miR-107	Dicer	−	Promotes neurogenesis	[189]
miR-381	Hes1	+	Promotes neural stem cell proliferation and differentiation	[80]
miR-765	Hes1	+	Promotes neural stem cell proliferation and differentiation	[72]
miR-106b~25 cluster	TGFβ insulin/IGF-FoxO	+	Promotes neural stem/progenitor cell proliferation and neuronal differentiation	[190]
Let-7 family	HNF4A	−	Promotes neuroblast proliferation	[128]
			Self-renewal	[191]
miR-34a	MYCN	+	Promotes proliferation	[192]
miR-184	AKT2	−	Inhibits neuroblastoma cell survival	[193]
miR-302/367	Unknown	+	Reprogram cells into neurons	[194]
miR-181a	Unknown	+	Promotion of the generation of TH-positive neuron	[195]
miR-125b	Unknown	+	Promotion of the generation of TH-positive neuron	[195]

Legend: + increased expression; − decreased expression; TH Tyrosine Hydroxylase.
